# Altered cardiac structure and function in newly diagnosed people living with HIV: a prospective cardiovascular magnetic resonance study after the initiation of antiretroviral treatment

**DOI:** 10.1007/s10554-022-02711-y

**Published:** 2022-08-26

**Authors:** Pieter-Paul S. Robbertse, Anton F. Doubell, Jan Steyn, Carl J. Lombard, Mohammed A. Talle, Philip G. Herbst

**Affiliations:** 1 Division of Cardiology, Department of Medicine, Faculty of Medicine and Health Sciences, Stellenbosch University and Tygerberg Hospital, PO Box 19063, Tygerberg, 7505 South Africa; 2 University of Pittsburgh HIV-Comorbidities Research Training Programme in South Africa, Pittsburgh, USA; 3 Biostatistics Unit, South African Medical Research Council, Cape Town, South Africa; 4 Division of Epidemiology and Biostatistics, Department of Global Health, Stellenbosch University, Cape Town, South Africa; 5 Department of Medicine, Faculty of Clinical Sciences, College of Medical Sciences, University of Maiduguri, Maiduguri, Nigeria

**Keywords:** HIV associated cardiomyopathy, Cardiovascular magnetic resonance, Cardiac function, Cardiac morphology, Pericardial effusion, Prospective

## Abstract

HIV associated cardiomyopathy (HIVAC) is a poorly understood entity that may progress along a continuum. We evaluated a group of persons newly diagnosed with HIV and studied the evolution of cardiac abnormalities after ART initiation. We recruited a group of newly diagnosed, ART naïve persons with HIV and a healthy, HIV uninfected group. Participants underwent comprehensive cardiovascular evaluation, including cardiovascular magnetic resonance imaging. The HIV group was started on ART and re-evaluated 9 months later. The cardiovascular parameters of the study groups were compared at diagnosis and after 9 months. The ART naïve group’s (n = 66) left- and right end diastolic volume indexed for height were larger compared with controls (n = 22) (p < 0.03). The left ventricular mass indexed for height was larger in the naïve group compared with controls (p = 0.04). The ART naïve group had decreased left- and right ventricular ejection fraction (p < 0.03) and negative, non-linear associations with high HIV viral load (p = 0.02). The left ventricular size increased after 9 months (p = 0.04), while the systolic function remained unchanged. The HIV group had a high rate of non-resolving pericardial effusions. HIV infected persons demonstrate structurally and functionally altered ventricles at diagnosis. High HIV viral load was associated with left- and right ventricular dysfunction. Cardiac parameters and pericardial effusion prevalence did not show improvement with ART. Conversely, a concerning trend of increase was observed with left ventricular size. These subclinical cardiac abnormalities may represent a stage on the continuum of HIVAC that can progress to symptomatic disease if the causes are not identified and addressed.

**Supplementary Information:**

The online version contains supplementary material available at 10.1007/s10554-022-02711-y.

## Introduction

HIV infection is globally prevalent and is one of mankind’s most persistent epidemics. HIV is associated with cardiovascular dysfunction and death [1], even in the era of antiretroviral therapy (ART), cardiovascular disease (CVD) persists [2].

CVD in people living with HIV (PLWH) exists on a spectrum, may be highly heterogenous, and differ between persons on ART and those not on ART [3–6]. In sub-Saharan Africa, HIV-associated cardiomyopathy (HIVAC) remains a significant contributor to CVD [7] and may manifest as asymptomatic ventricular dysfunction [8]. It is hypothesised that subclinical abnormalities represent early changes on a continuum toward dilated cardiomyopathy, given the persistence of appropriate driving factors [4]. It is not understood at which time point asymptomatic cardiovascular abnormalities become evident, but emerging evidence suggests that myocardial injury may occur as early as during acute HIV infection [9]. Contemporary evidence suggests that the hearts of newly infected persons with HIV may already be structurally and functionally abnormal [10–12].

Cardiovascular magnetic resonance imaging (CMR) is a powerful diagnostic modality and the most accurate tool to assess cardiac morphology and systolic function [13]. Utilising CMR, we investigated our hypothesis that HIVAC may be demonstrable as subtle structural and functional myocardial abnormalities at the time of HIV diagnosis. We prospectively evaluated the hearts of a contemporary HIV infected cohort before and after 9 months of ART treatment to evaluate the evolution of cardiac abnormalities on ART.

## Methods

### Study design and population

Newly diagnosed, HIV infected individuals (n = 66) were recruited into a prospective cohort study in South Africa from January 2020 to March 2022. Participants were recruited as out-patients at the time of testing positive for HIV. Age and sex matched, HIV uninfected controls (n = 22) were recruited from the same geographic areas. The study was approved by the Stellenbosch Human Research Ethics Committee (Ref S19/07/137) and all participants provided written informed consent. All participants recruited after March 2020 underwent severe acute respiratory syndrome coronavirus-2 PCR testing before inclusion. Inclusion criteria were age 18 to 55 years, no prior symptomatic heart disease, no ART prior to enrolment, no contraindication to CMR, not pregnant, and no acutely unwell patients (including current coronavirus disease-19). Persons with tuberculosis (TB) coinfection were not excluded. HIV/TB coinfected participants were enrolled at least 2 weeks after initiation on TB treatment while still ART naïve. All treatment and workup provided to HIV infected participants were in line with local and national treatment guidelines [14].

### Clinical data collection and follow up

Data was collected at three time points for HIV infected participants: A baseline visit prior to ART (naïve group), an interim clinical assessment visit (4 months following ART initiation), and at 9 months of ART (ART group). The control group was evaluated at a single time point.

Full anthropometric, biochemical, immunological, virological, electrocardiogram (ECG), echocardiographic and CMR investigations were performed at baseline and 9 months. Additional CD4 count and HIV viral load measurements were made at the interim visit. For the baseline and final visit, participants were seen in the morning following a 10 hour fast. The World Health Organisation (WHO) HIV clinical stage was documented [15]. Blood pressure was measured and mean arterial pressure was calculated [(1/3 × pulse pressure) + diastolic blood pressure] [16]. Height and weight were measured, and body mass index (BMI) was calculated. A 12-lead ECG was performed and the six minute walk distance was recorded [17]. Fasting blood samples were sent to the on-site National Health Laboratory Service for measurement of urea and electrolytes, glucose, blood lipids, full blood count, differential cell count, HIV enzyme-linked immunosorbent assay, high sensitivity C-reactive protein (hsCRP), flow cytometry based CD4- and CD8 count, and HIV-1 viral load. The linear range for the measurement of HIV viral load by our core laboratory is 20 to 10,000,000 copies/ml or 1.3 to 7 log (Abbot Alinity M HIV-1 assay). Viral load reported by the laboratory as “lower than detectable level” were captured as 20 copies/ml (1.3 log). We defined persistent viraemia as a viral load of > 200 copies/ml in persons on ART at interim follow up or 9 months. In accordance with local clinical practice, gender-specific estimated glomerular filtration rate (eGFR) was calculated using the Cockcroft-Gault formula [14]. The HIV cohort was tested for SARS-CoV-2 immunoglobulin G antibodies at the 9 months visit.

### Cardiovascular magnetic resonance

#### Image acquisition

CMR studies were performed on a single 1.5-T magnetic resonance scanning system (Magnetom Avanto, Siemens Healthcare, Germany) with commercially available sequences. Baseline and follow up studies were acquired by the same operator using standard methods [18]. Breath-held, ECG gated, balanced steady-state free precession cine images were obtained for the assessment of cardiac mass, volume, function, and morphological evaluation. T1-weighted turbo spin echo sequences were included for pericardial assessment.

#### Image analysis

**Fig. 1:**
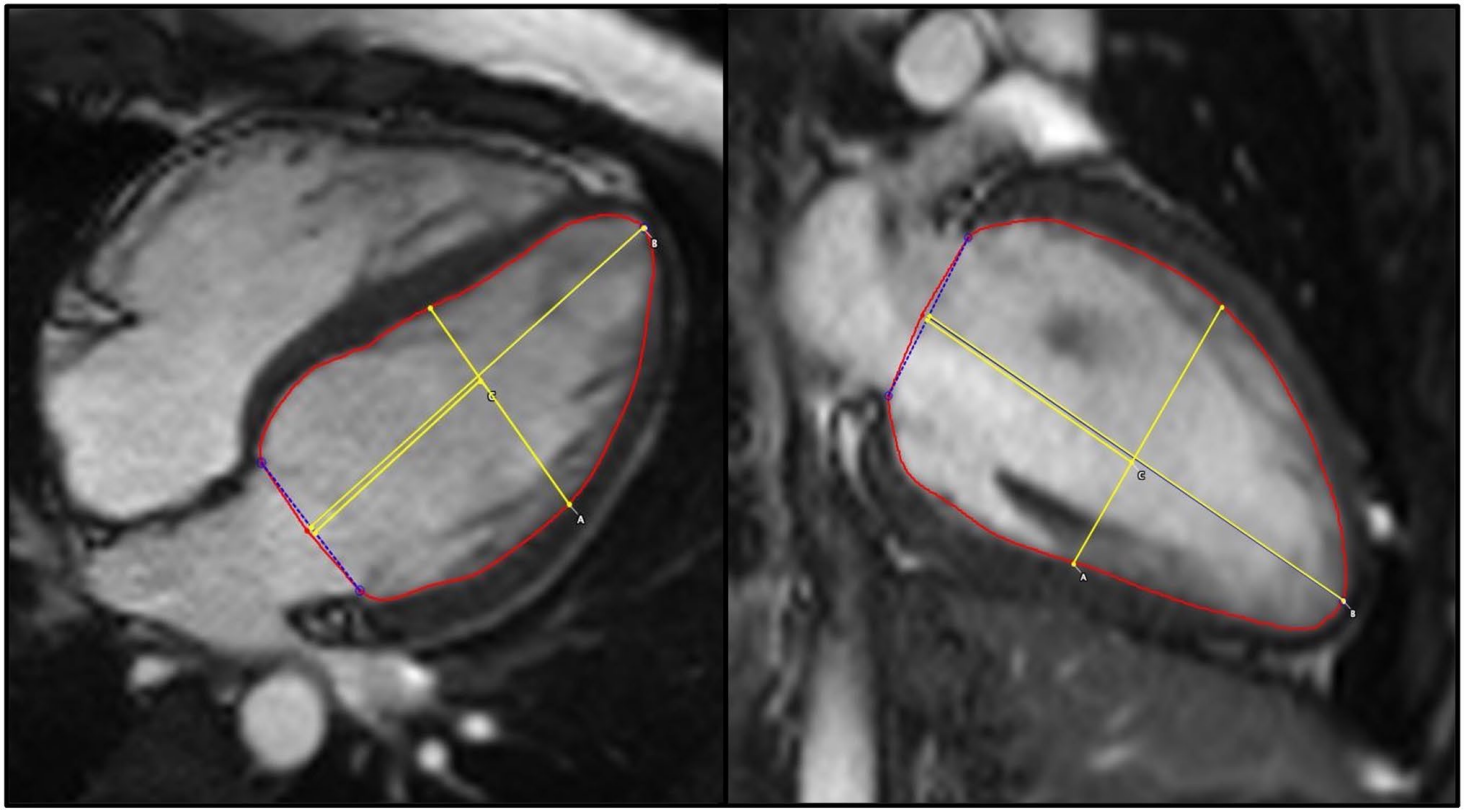
Measurements used to calculate the sphericity index in the 4 chamber (left) and 2 chamber (right) imaging planes

PSR performed unblinded analysis of all the CMR data using commercially available software [cvi42, Circle Cardiovascular Imaging, version 5.11.2 (1497)]. Semi-automated, artificial intelligence assisted endocardial and epicardial left ventricular (LV) and right ventricular (RV) borders were traced at end-systole and end-diastole to determine LV volumes and mass. Papillary muscles were excluded from the LV blood pool. Body surface area was calculated using the Mosteller formula [square root of (height (cm) x weight (kg)/3600)]. LV wall thickness was measured at the heart base in short axis. The LV sphericity index (SI) was calculated to evaluate the LV geometry [19] as follows: Midventricular length (A) divided by longitudinal length (B) (Fig. 1). The average SI from these two imaging planes were calculated. The midventricular level was determined by halving the measurement from the middle of the annular plane to the apex.

MAT re-read 10% of the cohort (n = 15) in a random and blinded fashion to assess the inter-reader variability within the study. PGH supervised the CMR analysis and reviewed key findings and measurements. He was blinded to the clinical information. PSR and MAT both have 3 years and PGH has 7 years’ experience in formal CMR analysis.

### Pulmonary pressure estimation

A tricuspid regurgitation (TR) doppler trace was acquired in the left lateral decubitus position [20]. The right atrial (RA) pressure was estimated using the inferior vena cava diameter and collapsibility during sniff and systolic pulmonary artery pressure was calculated as 4 x (maximum TR velocity)^2^ + estimated RA pressure [21].

### Statistical analysis

Sample size was calculated for CMR-measured LV volumes, LV mass and LV ejection fraction (LVEF) using paired samples *t*-test for the prospective evaluation of the HIV infected group. Statistical analyses were performed using SPSS version 27 (IBM corporation, New York) and STATA version 17.0 (Stata Corporation, USA). Continuous data are presented as mean ± standard deviation. For non-normally distributed data, the median and interquartile ranges (IQR) are stated. Categorical data is presented as frequencies with percentages. The normality of data was tested using the Shapiro–Wilk test. For cross sectional analyses between the HIV infected and control group, the chi-square test, independent samples *t*-test, or Mann–Whitney *U* test was used as appropriate. For prospective analyses of the HIV infected group, the paired samples *t*-test or Wilcoxon signed-rank test was employed as appropriate. Inter-reader reliability on quantitative data was assessed using interclass correlation coefficient (ICC), using two-way random, average measurements with absolute agreement. Bivariate correlations were calculated using Spearman’s Rho test. To assess specific non-parametric associations, a fractional polynomial regression model was constructed. Multivariate linear regression models were constructed to adjust key CMR parameters [LV end diastolic volume (LVEDV), right ventricular end diastolic volume (RVEDV), LV mass, LVEF and RVEF] for possible confounding factors between study groups. Statistical significance was 2-tailed and defined as a p value ≤ 0.05.

## Results

### Study group characteristics

The study group’s demographics and clinical data are shown in Table 1. The HIV infected group and controls were well matched in terms of age and sex. Both groups were relatively young with a shared mean age of 33 years. The socio-economic status of the HIV infected persons was lower than the control group. Almost half of the HIV group earned less than R5000 (about 340 United States dollars at the time of writing) per month. Compared to controls, the naïve group showed a trend to have more smokers (27% vs 50%; p = 0.06). Ethanol use was statistically similar in the naïve group and controls (p = 0.2). Notably, the ART group tended to consume less alcohol after 9 months on ART (p = 0.03).

**Table 1:** Study population

Parameter	Control group (n = 22)	HIV infected ART naïve (n = 66) (Naïve)	p value^a^	HIV infected 9 months on ART (n = 66) (ART)	p value^b^
Age (years)	33 ± 7	33 ± 8	0.9	–	–
Female sex	11 (50%)	30 (46%)	0.8	–	–
Self–identified race					
Black African	9 (41%)	53 (80%)	**0.002**	–	–
Coloured	12 (55%)	12 (18%)			
Caucasian	1 (4%)	1 (2%)			
Monthly household income (South African Rand per month)					
< 5000	4 (18%)	30 (45%)	**0.003**	–	–
5000 to 20,000	14 (77%)	35 (53%)			
> 20 000	4 (18%)	1 (2%)			
Smoking history	6 (27%)	33 (50%)	0.06	34 (52%)	–
Ethanol (units per week)	0.5 (0 to 7)	4 (0 to 12)	0.2	0 (0 to 11)	**0.03**
Waist circumference (cm)	95 ± 18	80 ± 9	** < 0.001**	82 ± 9	0.09
Body mass index (kg/m^2^)	30 ± 8	23 ± 4	**0.001**	24 ± 4	**0.03**
World Health Organisation HIV clinical stage					
I	–	26 (39%)	–	26 (39%)	–
II		19 (29%)		19 (29%)	
III		20 (30%)		19 (29%)	
IV		1 (2%)		2 (3%)	
On treatment for TB		10 (15%)	–	2* (3%)	
Pulmonary	–	8		0	
Extra–pulmonary		2		2	
Days since HIV diagnosis	–	8 (4 to 22)	–	302 (283 to 386)	
Time to interim follow up (months)	–	–		4 (3 to 7)	–
Time to 9 months follow up (months)	–	–		9 (9 to 10)	–
History of proven COVID–19 (mild disease)	1 (5%)	None	–	1 (2%)	–
Antibody evidence of SARS–CoV–2 infection in unimmunised persons	None	Not performed at baseline	-	17 (26%)	–
Medications					
Salbutamol metered dose inhaler	1 (5%)	1 (2%)	–	1 (2%)	–
Rifampicin/isoniazid/pyrazinamide/ethambutol	–	10 (15%)		2 (3%)	
Trimethoprim/sulphametoxazole	–	21 (32%)		13 (20%)	
Isoniazid prophylaxis	–	1 (2%)		36 (55%)	
Pyridoxine	–	10 (15%)		38 (56%)	
Tenofovir/lamivudine/dolutegravir	–	Naïve		64 (97%)	
Losartan		–		2 (3%)	
Amlodipine/hydrochlorothiazide	–	1 (2%)		3 (5%)	
Statins	–	–		1 (2%)	
Clinical course on ART					
Immunological failure^**^ at interim or final follow up	–	–	–	13 (20%)	–
Viral load > 200 copies/ml at interim or 9 months	–	–	–	15 (23%)	–
6 min walk test distance (m)	637 ± 84	619 ± 95	0.4	622 ± 73	0.8

*HIV* Human immunodeficiency virus, *TB* tuberculous disease, *COVID-19* coronavirus disease-19, *SARS-CoV 2* severe acute respiratory syndrome coronavirus-2, *ART* antiretroviral therapy

^*^One additional patient developed tuberculosis after recruitment

^**^Failure was defined as a CD4 drop to below baseline or a 50% decrease from the on-treatment peak value

^a^Controls vs naïve

^b^Naïve vs ART

Bold values indicates p value < or = 0.05

Chronic diseases and medication use were uncommon. Only two HIV positive participants had a known diagnosis of hypertension and during follow up, one HIV infected participant developed hypertension and was placed on treatment. Metabolic syndrome was rarely encountered, with only one control and two participants fulfilling criteria for metabolic syndrome as defined by the National Cholesterol Education Program Adult Treatment Panel III [22] at enrolment.

### The general health of the HIV infected group

More than two thirds of the cohort had early (WHO I or stage II) disease. A third of the participants visited their health centre due to significant, unexplained weight loss and were otherwise well. The remainder of the HIV group presented due to their partner testing positive or with HIV related complications and included dermatological manifestations of HIV, lymphadenopathy, respiratory-, gastrointestinal-, and miscellaneous complaints. HIV/TB coinfection was found to be prevalent among the naïve group. Ten naïve participants (15% of the HIV group) had TB disease diagnosed at enrolment. All but one of the TB cases were mild and managed as out-patients. Extrapulmonary TB was diagnosed incidentally in 2 cases at baseline (abdominal and pleural TB respectively). No HIV infected participant reported subjective loss of functional capacity or dyspnoea. Furthermore, the 6-min walk distance demonstrated a comparable functional status between the naïve group and controls (619 vs 637 m; p = 0.4).

The median time from HIV diagnosis to the first research visit was 8 days.

The biochemical and immunological data collected are shown in Table 2. The median CD4 count for the naïve group was 287 cells/μl (IQR: 181 to 382). As expected, the CD4 count increased after initiation on ART and measured 347 cells/μl (IQR: 231–576) at interim follow up and 375 cells/μl (IQR: 268–589) at 9 months. At 9 months on ART, participants’ median viral load measured < 20 copies/ml (IQR: < 20–36).

**Table 2:** Biochemical and immunological data

Parameter	Control group (n = 22)	HIV infected ART naïve (n = 66) (Naïve)	p value^a^	HIV infected 9 months on ART (n = 66) (ART)	p value^b^
Serum creatinine (μmol/l)	72 ± 14	70 ± 14	0.5	82 ± 16	** < 0.001**
Estimated glomerular filtration rate (ml/min/1.73 m^2^)	111 ± 38	92 ± 22	**0.03**	82 ± 19	** < 0.001**
Fasting blood glucose (mmol/l)	5.0 ± 0.6	4.5 ± 0.5	** < 0.001**	4.7 ± 0.6	**0.002**
Haematocrit (%)	42 ± 4	39 ± 7	**0.02**	41 ± 5	**0.003**
Total fasting serum cholesterol (mmol/l)	4.3 (3.9–4.9)	3.4 (3.0–3.9)	** < 0.001**	3.5 (3.1–4.1)	**0.04**
Triglycerides (mmol/l)	0.9 (0.6–1.2)	0.9 (0.7–1.2)	0.9	0.7 (0.6–1.0)	**0.03**
HDL cholesterol (mmol/l)	1.4 (1.2–1.5)	1.0 (0.8–1.3)	** < 0.001**	1.2 (1.0–1.4)	** < 0.001**
LDL cholesterol (mmol/l)	2.7 (2.0–3.1)	1.8 (1.6–2.4)	**0.005**	2.0 (1.6–2.4)	0.6
High sensitivity C-reactive protein (mg/l)	2.4 (1.1–9.2)	3.6 (0.9–13.2)	0.5	1.9 (0.5–7.2)	**0.01**
White cell count (10^9^ cells/l)	5.8 (4.5–7.9)	5.1 (3.9–6.2)	**0.02**	5.1 (3.9–6.4)	0.2
Lymphocyte count (10^9^ cells/l)	2.0 (1.7–17.9)	1.4 (1.1–2.0)	** < 0.001**	1.4 (1.2–1.8)	0.9
Monocyte count (10^9^ cells/l)	0.4 (0.3–3.4)	0.3 (0.2–0.4)	**0.05**	0.3 (0.2–0.4)	0.7
Eosinophil count (10^9^ cells/l)	0.1 (0.1–1.1)	0.1 (0.1–0.3)	0.4	0.1 (0.1–0.2)	0.06
CD4 count (cells/μl)	–	287 (181–382)	–	375 (268–589)	** < 0.001**
CD8 count (cells/μl)	–	804 (570–1029)	–	608 (462–783)	** < 0.001**
CD4:CD8 ratio	–	0.35 (0.23–0.46)	–	0.61 (0.40–0.97)	** < 0.001**
CD4:CD8 ratio < 1	–	61 (92%)	–	48 (73%)	**0.002**
HIV viral load (copies/ml)		81 525 (9781–320 497)	–	20* (20–36)	** < 0.001**
HIV viral load (log copies/ml)	–	4.9 (4.0 – 5.5)	–	1.3** (1.3 –1.6)	** < 0.001**

*HDL* high density lipoprotein, *LDL* low density lipoprotein, *HIV* Human immunodeficiency virus

^*^20 copies/ml (log = 1.3) is the lower limit of measurement for our HIV–1 assay

^a^Controls vs naïve

^b^Naïve vs ART

Bold values indicates p value < or = 0.05

Despite their preserved functional status, the naïve group’s anthropometry and biochemical evaluation differed considerably from the control group: The naïve group’s mean BMI was considerably lower compared with controls (23 vs 30 kg/m^2^; p = 0.001). Waist circumference was on average 15 cm lower compared to the control group (80 vs 95 cm; p =  < 0.001). The eGFR, fasting blood glucose, haematocrit, total fasting serum cholesterol, HDL cholesterol, LDL cholesterol, white cell count, lymphocyte count, and monocyte count were all significantly lower compared with controls (p ≤ 0.03). eGFR was 21% higher in controls compared to the naïve group (111 vs 92 ml/min/1.73m^2^; p < 0.001). The median hsCRP measured 50% higher in the naïve group compared with controls (3.6 vs 2.4 mg/l), but did not reach statistical significance (p = 0.5). A marked decrease in the median hsCRP was observed when comparing the naïve and ART groups (3.6–1.9 mg/l; p = 0.01).

### Cardiac morphology and function

The morphological and functional data is shown in Table 3. Pericardial effusions were considerably more prevalent in the naïve group when compared with controls (67 vs 18%, p < 0.001). The effusions were small (< 10 mm), haemodynamically insignificant, and not associated with TB disease. The prevalence of pericardial effusion did not change significantly after 9 months on ART (p = 0.9). No person in the cohort had a TR velocity greater than 2.8 m/s or estimated RA pressures greater than 5 mmHg. On average, the TR velocity in the naïve group was 26% higher compared with controls (p = 0.003). No significant change in the TR velocity was observed after the initiation of ART (p = 0.2). When comparing the TR velocity in smokers and non-smokers, and persons with and without TB, no difference is statistically evident in the naïve group (2.04 vs 1.93 m/s, p = 0.2 & 1.93 vs 1.99 m/s, p = 0.7).

**Table 3:** Cardiac function and morphology

Parameter	Control group (n = 22)	HIV infected ART naïve (n = 66) (Naïve)	p value^a^	HIV infected 9 months on ART (n = 66) (ART)	p value^b^
Resting heart rate on electrocardiogram	70 ± 10	73 ± 15	0.3	65 ± 13	** < 0.001**
Heart rhythm					
Sinus rhythm	19 (86%)	55 (83%)	0.9	50 (76%)	0.06
Sinus bradycardia	3 (14%)	7 (11%)		16 (24%)	
Sinus tachycardia	None	4 (6%)		None	
Systolic blood pressure (mmHg)	119 ± 13	114 ± 15	0.2	112 ± 14	0.4
Diastolic blood pressure (mmHg)	76 ± 9	72 ± 10	0.08	69 ± 10	**0.03**
Pulse pressure (mmHg)	43 ± 10	42 ± 9	0.7	43 ± 10	0.6
Mean arterial pressure (mmHg)	90 ± 9	86 ± 11	0.09	84 ± 10	0.1
Maximum TR velocity (m/s)	1.55 ± 0.4	1.95 ± 0.5	**0.003**	2.01 ± 0.5	0.2
Estimated pulmonary pressure (mmHg)	16 ± 7	21 ± 7	**0.008**	22 ± 7	0.2
Cardiac magnetic resonance imaging					
Presence of pericardial effusion	4 (18%)	44 (67%)	** < 0.001**	43 (65%)	0.9
Thickest LV segment					
Anterior septum	17 (77%)	55 (83%)	1.0	55 (83%)	1.0
Inferior septum	4 (18%)	10 (15%)		10 (15%)	
Other	1 (5%)	1 (2%)		1 (2%)	
Maximum LV wall thickness (mm)	9.1 ± 1.5	9.3 ± 1.4	0.5	9.5 ± 1.4	0.09
Sphericity index in 2 chamber	0.56 ± 0.05	0.55 ± 0.05	0.6	0.54 ± 0.04	0.07
Sphericity index in 4 chamber	0.50 ± 0.05	0.50 ± 0.05	0.7	0.50 ± 0.05	0.4
Averaged sphericity index	0.53 ± 0.04	0.53 ± 0.05	0.7	0.52 ± 0.04	0.3
LA diameter (mm)	33 ± 5	30 ± 4	**0.03**	30 ± 4	1.0
LA area (cm^2^)	20 ± 3	20 ± 4	0.8	21 ± 3	0.2
RA area (cm^2^)	19 ± 3	20 ± 4	0.4	20 ± 3	0.06
LV end diastolic volume (ml)	133 ± 24	143 ± 30	0.2	148 ± 28	**0.05**
LV end diastolic volume indexed to height (ml/m)	81 ± 13	86 ± 16	0.03^*^	89 ± 15	**0.04**
LV end diastolic volume indexed to body surface area (ml/m^2^)	71 ± 13	84 ± 15	** < 0.001**	86 ± 14	0.3
LV end systolic volume indexed to body surface area (ml/m^2^)	27 ± 8	34 ± 9	** < 0.001**	35 ± 8	0.5
RV end diastolic volume (ml)	121 ± 37	138 ± 29	**0.03**	140 ± 29	0.4
RV end diastolic volume indexed to height (ml/m)	73 ± 21	83 ± 16	0.02^*^	84 ± 16	0.4
RV end diastolic volume indexed to body surface area (ml/m^2^)	68 ± 14	82 ± 15	** < 0.001**	81 ± 14	0.6
RV end systolic volume indexed to body surface area (ml/m^2^)	25 ± 8	33 ± 8	** < 0.001**	32 ± 9	0.5
LV mass (g)	97 (84 to 115)	102 (86 to 126)	0.5	103 (84 to 127)	0.9
LV mass indexed to body surface area (g/m^2^)	55 (46 tot 58)	63 (52 to 71)	**0.003**	60 (51 to 71)	0.2
LV mass indexed to height (g/m)	60 (51 to 71)	63 (53 – 74)	0.04^*^	63 (53 – 75)	0.9
LV ejection fraction (%)	63 ± 5	59 ± 6	0.03^*^	59 ± 5	0.9
LV stroke volume (ml)	84 ± 15	85 ± 17	0.8	88 ± 16	**0.05**
RV ejection fraction (%)	64 ± 5	60 ± 5	0.05^*^	62 ± 6	0.09
RV stroke volume (ml)	80 ± 14	83 ± 17	0.5	86 ± 17	0.08

*TR* tricuspid regurgitation, *LA* left atrium, *RA* right atrium, *LV* left ventricle, *RV* right ventricle

*Adjusted for covariates

^a^Controls vs naïve

^b^Naïve vs ART

Bold values indicates p value < or = 0.05

#### Cardiac chamber morphology

No difference in the left atrial (LA) and RA area was seen between the study groups. Although, the naïve group’s LVEDV indexed to height did not demonstrate a statistically significant difference compared to controls (81 ml vs 86 ml/m; p = 0.2), with correction for covariates, a significant difference was evident (p = 0.03) and is elaborated upon in multivariate analysis section. After 9 months of ART, the LVEDV indexed to height of the HIV group increased further (86 to 89 ml/m; p = 0.04). Comparing the control with the ART group, the indexed LVEDV measured 10% larger in the ART group reflecting a mean difference of 8 ml/m (p = 0.02). This translates to a mean difference of 15 ml in the non-indexed LVEDV between the control and ART group (133 vs 148 ml; p = 0.03).

The RVEDV indexed to height demonstrated larger RVs in the naïve group compared with controls (83 vs 73 ml/m, p = 0.03). However, the indexed and non-indexed RVEDV in the ART group did not change significantly from baseline (p > 0.4).

A median difference of 3 g/m in the LV mass indexed to height was demonstrable between the naïve and control group. This unadjusted value was however not statistically significantly different and no change on ART was observed.

The maximum LV wall thickness, thickest LV segment distribution, SI, LA area, and RA area did not demonstrate significant differences between the three groups studies (p values > 0.07).

#### Ventricular function

Both the LVEF and RV ejection fraction (RVEF) were on average 4% lower in the naïve group compared with controls (p = 0.03 and 0.02 respectively). After 9 months on ART, no change in either LVEF or RVEF was measured (p = 0.09).

The LV and RV stroke volumes were similar in the control and naïve group. The LV stroke volume increased by 3 ml in the HIV group after 9 months on ART (p = 0.05).

#### CMR findings in a subgroup with persistent viraemia

Persistent viraemia was observed in 15 persons (23%) from the HIV group. All these persons had a history of suboptimal ART compliance. Prospective parameters of the group are shown in Table 4. The findings in this group were found to be similar to the larger cohort in that CMR parameters appear unchanged after 9 months. Similar to the main cohort, the LVEDV appeared to increase to the same degree over time (5 ml), but was not statistically significant due to the small sample size.

**Table 4:** Subgroup analysis of persons with viral load > 200 copies/ml after the initiation on ART

Parameter	Persistent viraemia group (n = 15) (Naïve)	Persistent viraemia group (n = 15) (ART)	p value
CD4 count (cells/μl)	277 (214–355)	388 (209 to 529)	**0.01**
CD8 count (cells/μl)	980 (774–1536)	671 (465 to 1018)	**0.009**
HIV viral load (copies/ml)	77 005 (7478–350 082)	2389 (20 to 89 339)	**0.005**
HIV viral load (log copies/ml)	4.9 (3.9–5.5)	3.4 (1.3*–5.0)	**0.001**
Presence of pericardial effusion	10 (67%)	11 (73%)	0.7
LA diameter (mm)	31 ± 5	32 ± 4	0.1
LA area (cm^2^)	19 ± 3	20 ± 3	0.2
RA area (cm^2^)	18 ± 4	19 ± 3	0.1
LV end diastolic volume (ml)	132 ± 30	137 ± 27	0.5
LV end diastolic volume indexed to body surface area (ml/m^2^)	81 ± 16	81 ± 14	1.0
RV end diastolic volume indexed to body surface area (ml/m^2^)	79 ± 17	78 ± 11	0.6
RV end diastolic volume (ml)	127 ± 28	132 ± 24	0.7
LV mass (g)	84 (74 to 128)	94 (79 to 117)	**0.02**
LV mass indexed to body surface area (g/m^2^)	59 (50 to 65)	60 (53 to 70)	**0.02**
LV ejection fraction (%)	59 ± 5	59 ± 5	0.2
LV stroke volume (ml)	79 ± 18	82 ± 16	0.1
RV ejection fraction (%)	60 ± 5	61 ± 5	0.1
RV stroke volume (ml)	78 ± 17	81 ± 15	0.3

*LV* left ventricle, *RV* right ventricle, *LA* left atrial, *RA* right atrial

*20 copies/ml (log = 1.3) is the lower limit of measurement for our HIV-1 assay

Bold values indicates p value < or = 0.05

#### Inter-reader reliability

The re-read quantitative parameters demonstrated good to excellent ICC between readers. The quantitative parameters analysed, and their correlations included: Left atrial area (ICC = 0.94), RA area (ICC = 0.96), LVEDV (ICC = 0.96), RVEDV (ICC = 0.97), LV mass (ICC = 0.84), LVEF (ICC = 0.77, p = 0.004), and RVEF (ICC = 0.70). Probability values were all calculated to be highly significant (p < 0.001).

### Bivariate analysis

**Fig. 2:**
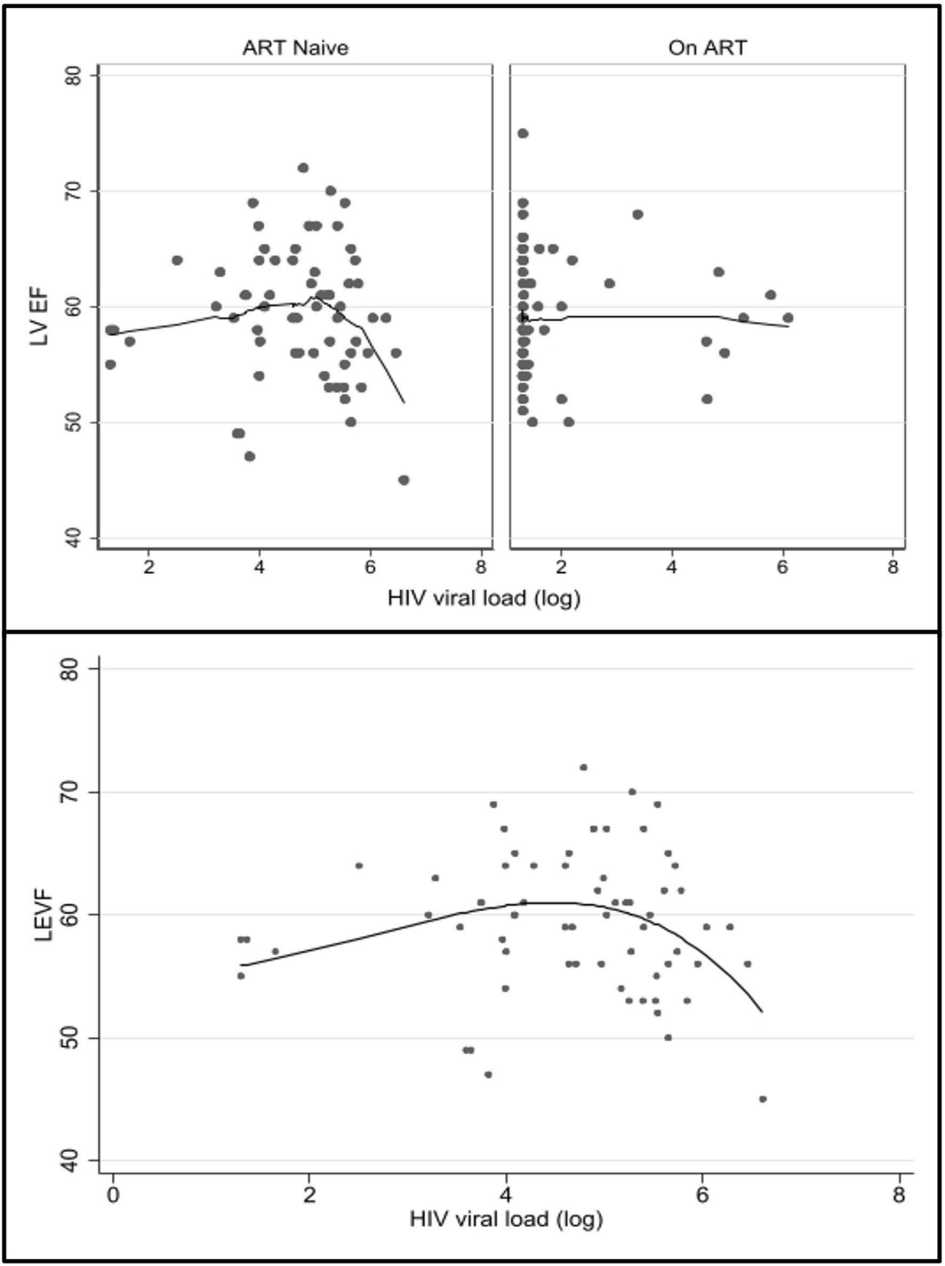
Top: Scatter plot with a fitted line using the Lowess smoothing demonstrating a negative, non-linear association between left ventricular ejection fraction (LVEF) and HIV viral load (log). Below: A fractional polynomial model of LVEF and HIV viral (log), demonstrating a decrease in the LVEF is evident at log 5, suggesting a high viral load decrease LVEF by an unknown mechanism (p = 0.02). A similar negative, non-linear association (not shown) was seen with right ventricular ejection fraction and HIV viral load (log) (p = 0.02)

A full list of significant bivariate correlations and notable non-correlations within the HIV infected group (132 data points) are shown in online appendix A. The bivariate analysis of LVEF, RVEF, LVEDV, RVEDV, and LV mass demonstrated few significant correlations. The Spearman’s Rho correlation between LVEF and RVEF, and HIV viral load was not significant. However, a significant negative, non-linear association was found in ART naïve persons when the viral load reaches a threshold of approximately log 5 (Fig. 2). This was significant in both LVEF and RVEF (p = 0.02). At 9 months on ART, this association is not demonstrable. No association between CD4 count and LVEF or RVEF was found.

### Multivariate analysis

Correlations from online appendix A and clinical differences in the control and ART naïve group were used to construct a multivariate linear regression model to adjust the LVEDV indexed to height, RVEDV indexed to height, LVEF, and RVEF. Covariates adjusted for were for age, sex, race, HIV status, ethanol use and current TB disease (see online appendix B, C, D and E). Due to a distinctive correlation profile, the LV mass for height was corrected for age, sex, race, HIV status, eGFR and current TB disease (see online appendix F). Comparing the control and naïve group, the mean differences between the above CMR parameters all proved to be significant after multivariate adjustment. The LVEF and RVEF were both 3% lower in the naïve group (p < 0.05). The LVEDV and RVEDV indexed to height were 7 and 9 ml/m larger in the naïve group compared with controls (p < 0.03). After adjustment, the LV mass indexed to height demonstrated ventricles that were on average 6 g/m heavier in the naïve group compared with controls (p = 0.04).

## Discussion

This study is the first to prospectively evaluate cardiac morphology and function in an African cohort of PLWH using CMR. Capitalising on CMR’s excellent inter- and intra-observer variability, we were able to prospectively detect subtle differences in PLWH after ART initiation. Furthermore, we were able to compare PLWH with a contemporary group of HIV uninfected individuals in a cross-sectional fashion. The study better represents women and persons living in sub-Saharan Africa. Our HIV infected participants were recruited at the time of HIV diagnosis and provided the rare opportunity to essentially “fingerprint” the cardiovascular systems of PLWH as they presented. This was done before the influence of ART and additional factors that make research increasingly challenging as time passes [4]. Our study groups were relatively young and essentially free from cardiovascular comorbidities. This high level of homogeneity allowed for more reliable comparisons and conclusions.

The key findings of our study include:

- We found altered systolic function of both ventricles in PLWH. This was already evident at the time of HIV diagnosis and a link with viral load above log 5 is demonstrable with LV and RV systolic function. The ventricular function remained static after 9 months on ART whether viral suppression was achieved or not.
- Secondly, the LV size and mass of ART naïve PLWH were larger than uninfected persons. Of concern, after the initiation of ART, the LV size showed a trend toward increase.
- Thirdly, the RV size was already demonstrably larger in the HIV group at the time of diagnosis. Changes in the RV function and morphology were not observed after 9 months of ART.
- Lastly, small pericardial effusions were highly prevalent in the HIV cohort. Despite 9 months of ART and significantly decreased systemic inflammation as reflected by the decrease in hsCRP, the pericardial effusions persisted.

### Subclinical HIVAC

In earlier CMR-work from our group, we evaluated patients with symptomatic HIVAC that were characterised by larger, thicker ventricles with impaired systolic function [4]. After the initiation of ART, the pathological imaging findings improved, but did not resolve. Our hypothesis of HIVAC developing on a continuum, already present during early HIV infection, is consistent with our study’s observations. The phenotype of symptomatic HIVAC compared with our asymptomatic cohort shows similarities in terms of function and morphology. After 9 months of ART, the cardiac function and morphology of PLWH did not exhibit a trend towards normality and rather suggest a static underlying myopathy. In the case of LVEDV, a worsening trend was observed. In the HIV group, we have demonstrated that high HIV viral load is associated with decreased ejection fraction at baseline, that does not normalise after viral suppression. It is tempting to speculate that this may be due to the direct cardiotoxic effects of HIV. However, many secondary effects from HIV infection are possible [2, 6, 23] and one should caution against the over-interpretation of this finding as proof of direct viral cardiotoxicity.

### Ventricular function

The decreased LV systolic function evident at the time of HIV diagnosis corresponds with research done outside of Africa. In keeping with our findings, two small CMR studies evaluating ART naïve PLWH found the LVEF to be, on average, 4% lower compared with uninfected persons [10, 12]. No prospective CMR research on ART naïve PLWH is available for comparison. However, prospective, echocardiographic findings correlate with our observations in that after 11 months of ART, no significant change in the echocardiographic abnormalities were observed [8]. Other than the tendency of LVEDV to increase, we did not observe significant changes in the other CMR parameters over time. The static nature of our study parameters deserve consideration. On the one hand it may indicate the positive influence of ART, in turn causing a decrease in systemic inflammation and the termination of detrimental biological processes that damage the myocardium. Another possibility may be the presence of irreversible myocardial injury, sustained during [9] or shortly after HIV seroconversion. Lastly, ART and its beneficial effects did not address or only partially addressed the detrimental biological processes [24] and changes are not readily apparent at 9 months. Given our findings in the explorative subgroup with persistent viraemia, this is the most likely scenario. Our findings reflect the short-term effects of ART on cardiovascular abnormalities, and unfortunately it remains unknown whether these parameters will change significantly in the long term. With longer follow up intervals in future research, appreciable changes may become increasingly apparent.

Our HIV cohort demonstrated decreased RV function. There is a paucity of literature regarding asymptomatic RV dysfunction, especially in the ART naïve subgroup. Available research suggests that this is likely a separate entity from LV dysfunction [25], but we observed a correlation between LV and RV systolic function in the HIV group. We considered the presence of pulmonary pathology as a possible explanation for this. However, no association with RVEF and current TB disease was demonstrable. HIV associated pulmonary arterial hypertension is an incompletely understood entity [6, 26] and was considered in our study. We did find evidence suggesting relatively increased pulmonary pressures in the HIV infected group that correlated with the RV size. This finding will require further study as it is not explained by causes other than HIV in the cohort.

### Ventricular morphology and the use of indexed cardiac values

After covariate adjustment, we found the LV mass in the naïve group to be higher compared to their uninfected peers. Given the absence of abnormal loading conditions in our cohort, this may be explained by subclinical myocardial oedema [10, 12].

Prior CMR research has found varying results related to LV mass in PLWH [10, 27–29]. This may partly be explained by considering the method of indexing used in these trials, particularly the practice of indexing parameters to body surface area. Indexing cardiac measurements to body surface area is common clinical practice and is frequently employed in cardiovascular research. However, even in its earliest stages, HIV presents as a wasting syndrome. Profound weight loss may be encountered with disease progression or complications. ART may further influence the body habitus by causing weight gain and changes in body fat distribution [30]. We argue that a practice of indexing to height rather than BSA is likely superior when comparing data between study populations. Once adult height has been reached and length remains unchanged, parameters will not be influenced by weight fluctuations which should make height-based indexing preferred in these populations.

### Pericardial effusions, inflammation, and immune dysregulation

In the pre-ART era, pericardial effusions were reported to be one of the most prevalent cardiovascular abnormalities. When visualised on echocardiography, it signifies a poorer prognosis compared to those without an effusion [31]. In the era of ART, it has been suggested that the status quo has changed, as pericardial effusions are infrequently encountered with echocardiography [32]. However, CMR is a more sensitive tool to diagnose even small pericardial effusions, the presence of which is postulated to be secondary to underlying inflammation [10]. The presence of pericardial effusions have been shown to greatly improve the sensitivity of CMR to diagnose myocarditis [33]. Subsequently, pericardial effusion has been adopted as a supportive finding for myocarditis in the updated Lake Louise criteria [34]. The high rate of pericardial effusions in our HIV infected groups is consistent with current CMR literature evaluating PLWH [10]. Despite improved general health and lower inflammatory markers, we observed persistence of pericardial effusions after 9 months of ART. The absence of cardiac failure and primary pericardial disease support that pericardial effusions in our cohort may be due to low grade cardiac inflammation. The persistence of pericardial effusions after the initiation of ART, hints to the possibility that effusions observed in current post-ART era literature have been present since shortly after HIV seroconversion. This would support the thinking that HIV induces long term, subclinical cardiovascular inflammation despite viral suppression. Longstanding inflammation, in turn, drives the increased cardiovascular morbidity observed in PLWH.

### Significance of asymptomatic myocardial abnormalities

It can be argued that the cardiac parameters of the HIV group was normal when compared to normal reference ranges employed in clinical practice [35]. However, given the structural and functional abnormalities were already evident at diagnosis, coupled with the trend toward abnormality in the case of LVEDV, our findings are reason for concern. PLWH will spend their entire lives with HIV infection, known to cause immune activation despite viral suppression [36]. It is reasonable to expect that these parameters, driven by ongoing inflammation and secondary cardiac insults, will continue to worsen and eventually lead to symptomatic HIVAC. Our immunological data demonstrate marked immune system abnormalities with median baseline CD4 counts of less than 300 cells/μl, altered lymphocyte and monocyte counts, and high rates of inversed CD4:CD8 ratios (ratio < 1). These parameters tended to improved, but did not normalise after 9 months on ART. This demonstrates altered immunological function in our cohort that has been implicated in in chronic immune activation [23, 24]. Furthermore, our findings correlate with observations made in the Initiation of Antiretroviral Therapy in Early Asymptomatic HIV Infection (START) trial. This body of work suggests that ART alone is not sufficient to fully address the long term cardiovascular risk in PLWH [37]. Further work to optimise the long-term CVD risk in PLWH will be of great importance.

### Limitations

Firstly, no tissue characterisation or histological data is presented in this publication. As decreased systolic function, differences in the ventricular morphology, and frequent pericardial effusions were observed, it would be important to determine if myocardial abnormalities are present at the ultrastructural level and whether these findings support the presence of cardiac inflammation. Due to unjustifiable procedural risk, histological sampling in asymptomatic persons is inappropriate. Multiparametric mapping with CMR will be the tool of choice to acquire “virtual histology” from asymptomatic participants in future work. HIV associated myocardial fibrosis and sudden cardiac death was recently discussed in an autopsy-series [38] and it needs to be determined when and under what circumstances myocardial fibrosis develops. Given the static nature of myocardial function after the initiation on ART, irreversible myocardial injury remains a possibility to explain our findings. It is likely that a combination of oedema and fibrosis is present in the ventricles of PLWH at diagnosis.

Secondly, recruitment and research in the presence of a second, globally present virus proved challenging. The possible confounding effects of COVID-19 related myocardial injury were considered. Our understanding of COVID-19 related cardiovascular disease is incomplete [39], but we argue the effects of COVID-19 in our cohort were likely negligible: Two participants had prior proven COVID-19. Both cases were mild, self-limiting, flu-like illnesses and did not require any intervention. We were able to prove prior SARS CoV-2 exposure in 17 (26%) of our HIV infected group, with one symptomatic case of COVID-19. The abnormalities demonstrated in our cohort correlate with pre-COVID CMR research.

Thirdly, as the main analysis and processing of the CMR images were not performed blinded, the risk of bias was unavoidable. To minimise this risk, two additional, blinded readers were involved to supervise, reach consensus on findings, and formally review cases. The analysis of a random, blinded sample demonstrated a high to near perfect level of correlation between quantitative parameters between readers. This supports a high level of reliability of our data and the likely negligible effect of the unblinded analysis.

Lastly, the true duration of HIV infection after HIV seroconversion, despite extensive history taking and clinical evaluation, remains an educated guess. This shortcoming is not unique to our work and has been discussed extensively in prior research [40]. In short: We used the first positive HIV test as an undisputable time point at which HIV was present. The subsequent duration of HIV infection can be calculated with high accuracy. In accordance with clinical experience and research, the cohort is an accurate representation of the average new diagnosis of HIV in South Africa [41].

## Conclusion

We found HIV infected persons, at the time of HIV diagnosis, had structurally and functionally altered ventricles compared with controls. This provides evidence that HIVAC may already be present in select individuals during early HIV infection, well before the influence of ART. This may be due to underlying myocardial inflammation, evidenced by the otherwise unexplained, high rate of non-resolving pericardial effusions. Despite viral suppression at 9 months, ART did not improve the underlying abnormalities. Conversely, a concerning trend toward abnormality was observed in the LV size. An association of high HIV viral load and worse ventricular function was demonstrated at diagnosis, suggesting high levels of HIV replication may contribute to myocardial dysfunction and remodelling. Tissue characterisation to probe the underlying pathological processes is required.

## Supplementary Information

Below is the link to the electronic supplementary material.

Supplementary file1 (DOCX 42 KB)

## References

[1] Cohen IS, Anderson DW, Virmani R, Reen BM, Macher AM, Sennesh J, DiLorenzo P, Redfield RR (1986). Congestive cardiomyopathy in association with the acquired immunodeficiency syndrome. N Engl J Med.

[2] Bloomfield GS, Leung C (2017). Cardiac disease associated with human immunodeficiency virus infection. Cardiol Clin.

[3] Hsue PY, Waters DD (2017). Heart failure in persons living with HIV infection. Curr Opin HIV AIDS.

[4] Robbertse PS, Doubell AF, Nachega JB, Herbst PG (2021). The hidden continuum of HIV- associated cardiomyopathy: a focussed review with case reports. SA Heart J.

[5] Dominick L, Midgley N, Swart L-M, Sprake D, Deshpande G, Laher I, Joseph D, Teer E, Essop MF (2020). HIV-related cardiovascular diseases: the search for a unifying hypothesis. Am J Physiol Circ Physiol.

[6] Manga P, McCutcheon K, Tsabedze N, Vachiat A, Zachariah D (2017). HIV and nonischemic heart disease. J Am Coll Cardiol.

[7] Agbor VN, Essouma M, Ntusi NAB, Nyaga UF, Bigna JJ, Noubiap JJ (2018). Heart failure in sub-Saharan Africa: a contemporaneous systematic review and meta-analysis. Int J Cardiol.

[8] Luo L, Zeng Y, Li T, Lv W, Wang H, Guo F, Han Y, Xie J, Qiu Z, Li Y (2014). Prospective echocardiographic assessment of cardiac structure and function in Chinese persons living with HIV. Clin Infect Dis.

[9] Schuster C, Mayer FJ, Wohlfahrt C, Marculescu R, Skoll M, Strassl R, Pavo N, Popow-Kraupp T, Hülsmann M, Bauer M (2018). Acute HIV infection results in subclinical inflammatory cardiomyopathy. J Infect Dis.

[10] Ntusi N, O’Dwyer E, Dorrell L, Wainwright E, Piechnik S, Clutton G, Hancock G, Ferreira V, Cox P, Badri M (2016). HIV-1–related cardiovascular disease is associated with chronic inflammation, frequent pericardial effusions, and probable myocardial EDEMA. Circulation Cardiovasc Imaging.

[11] Robbertse P, Doubell A, Herbst P (2021). Cardiovascular magnetic resonance imaging reveals asymptomatic cardiomyopathy in newly diagnosed, HIV-infected South African adults. Eur Heart J Cardiovasc Imaging.

[12] Menacho K, Seraphim A, Ramirez S, Falcon L, Bhuva A, Alave J, Banda C, Mejia F, Salazar D, Putri A (2020). Myocardial inflammation and edema in people living with human immunodeficiency virus. JACC Cardiovasc Imaging.

[13] Grothues F, Smith GC, Moon JCC, Bellenger NG, Collins P, Klein HU, Pennell DJ (2002). Comparison of interstudy reproducibility of cardiovascular magnetic resonance with two-dimensional echocardiography in normal subjects and in patients with heart failure or left ventricular hypertrophy. Am J Cardiol.

[14] Western Cape Department of Health. The Western Cape Consolidated Guidelines for HIV Treatment: Prevention of Mother-to-Child Transmission of HIV (PMTCT), Children, Adolescents and Adults. 2020; 1–72. https://www.westerncape.gov.za/assets/departments/health/hiv_guidelines_16012020.pdf. Accessed 14 June 2022

[15] World Health Organisation. Annexure 1: WHO clinical staging of HIV disease in adults, adolescents and children. WHO case definitions of HIV for surveillance and revised clinical staging and immunological classification of HIV-related disease in adults and children. 2016; 1-2. https://apps.who.int/iris/bitstream/handle/10665/208825/9789241549684_eng.pdf;jsessionid=AB3DE3EDBC03C8D41CB21739CC7769C0?sequence=1. Accessed 14 June 2022

[16] Papaioannou TG, Protogerou AD, Vrachatis D, Konstantonis G, Aissopou E, Argyris A, Nasothimiou E, Gialafos EJ, Karamanou M, Tousoulis D (2016). Mean arterial pressure values calculated using seven different methods and their associations with target organ deterioration in a single-center study of 1878 individuals. Hypertens Res.

[17] The American Thoracic Society (2002). ATS Statement : guidelines for the six-minute walk test. Am J Respir Crit Care Med.

[18] Kramer CM, Barkhausen J, Bucciarelli-Ducci C, Flamm SD, Kim RJ, Nagel E (2020). Standardized cardiovascular magnetic resonance imaging (CMR) protocols: 2020 update. J Cardiovasc Magn Reson.

[19] Khanna S, Bhat A, Chen HH, Tan JWA, Gan GCH, Tan TC (2020). Left Ventricular Sphericity Index is a reproducible bedside echocardiographic measure of geometric change between acute phase Takotsubo’s syndrome and acute anterior myocardial infarction. IJC Heart Vasc.

[20] Masani N, Wharton G, Allen J, Chambers J, Graham J, Jones R, Rana B, Steeds R. Echocardiography: Guidelines for Chamber Quantification British Society of Echocardiography Education Committee [Internet]. https://www.bsecho.org/media/40506/chamber-final-2011_2_.pdf. Accessed 14 June 2022

[21] Augustine DX, Coates-Bradshaw LD, Willis J, Harkness A, Ring L, Grapsa J, Coghlan G, Kaye N, Oxborough D, Robinson S (2018). Echocardiographic assessment of pulmonary hypertension: a guideline protocol from the British Society of Echocardiography. Echo Res Pract.

[22] Third Report of the National Cholesterol Education Program (NCEP) (2002). Expert panel on detection, evaluation, and treatment of high blood cholesterol in adults (Adult Treatment Panel III) Final Report. Circulation.

[23] Bloomfield GS, Alenezi F, Barasa FA, Lumsden RBS, Mayosi BMVE (2016). Human immunodeficiency virus and heart failure in low- and middle-income countries. JACC Heart Fail.

[24] Currie PF, Boon NA (2003). Immunopathogenesis of HIV-related heart muscle disease. AIDS.

[25] Simon MA, Lacomis CD, George MP, Kessinger C, Weinman R, McMahon D, Gladwin MT, Champion HC, Morris A (2014). Isolated right ventricular dysfunction in patients with human immunodeficiency virus. J Card Fail.

[26] Sood V, Jermy S, Saad H, Samuels P, Moosa S, Ntusi N (2017). Review of cardiovascular magnetic resonance in human immunodeficiency virus-associated cardiovascular disease. S Afr J Radiol.

[27] Hsue PY, Hunt PW, Ho JE, Farah HH, Schnell A, Hoh R, Martin JN, Deeks SG, Bolger AF (2010). Impact of HIV infection on diastolic function and left ventricular mass. Circulation Heart Fail.

[28] Ntusi NAB, Ntsekhe M (2016). Human immunodeficiency virus-associated heart failure in sub-Saharan Africa: evolution in the epidemiology, pathophysiology, and clinical manifestations in the antiretroviral era. ESC Heart Fail.

[29] Chew KW, Liu CY, Ambale-Venkatesh B, Liao D, Horwich TB, Lima JAC, Bluemke DA, Paul Finn J, Butt AA, Currier JS (2017). Subclinical myocardial disease by cardiac magnetic resonance imaging and spectroscopy in healthy HIV/Hepatitis C virus-coinfected persons. J Int Med Res.

[30] Ballocca F, Gili S, D’Ascenzo F, Marra WG, Cannillo M, Calcagno A, Bonora S, Flammer A, Coppola J, Moretti C (2016). HIV infection and primary prevention of cardiovascular disease: lights and shadows in the HAART Era. Prog Cardiovasc Dis.

[31] Heidenreich PA, Eisenberg MJ, Kee LL, Somelofski CA, Hollander H, Schiller NB, Cheitlin MD (1995). Pericardial effusion in AIDS. Circulation.

[32] Lind A, Reinsch N, Neuhaus K, Esser S, Brockmeyer N, Potthoff A, Pankuweit S, Erbel R, Maisch B, Neumann T (2011). Pericardial effusion of HIV-infected patients - results of a prospective multicenter cohort study in the era of antiretroviral therapy. Eur J Med Res.

[33] Ong P, Athansiadis A, Hill S, Kispert E-M, Borgulya G, Klingel K, Kandolf R, Sechtem U, Mahrholdt H (2011). Usefulness of pericardial effusion as new diagnostic criterion for noninvasive detection of myocarditis. Am J Cardiol.

[34] Ferreira VM, Schulz-Menger J, Holmvang G, Kramer CM, Carbone I, Sechtem U, Kindermann I, Gutberlet M, Cooper LT, Liu P (2018). Cardiovascular magnetic resonance in nonischemic myocardial inflammation: expert recommendations. JACC.

[35] Kawel-Boehm N, Maceira A, Valsangiacomo-Buechel ER, Vogel-Claussen J, Turkbey EB, Williams R, Plein S, Tee M, Eng J, Bluemke DA (2015). Normal values for cardiovascular magnetic resonance in adults and children. J Cardiovasc Magn Reson.

[36] Neuhaus J, Jacobs DR, Baker JV, Calmy A, Duprez D, La Rosa A, Kuller LH, Pett SL, Ristola M, Ross MJ (2010). Markers of inflammation, coagulation, and renal function are elevated in adults with HIV infection. J Infect Dis.

[37] Siedner MJ (2016). START or SMART? timing of antiretroviral therapy initiation and cardiovascular risk for people with human immunodeficiency virus infection. Open Forum Infect Dis.

[38] Tseng ZH, Moffatt E, Kim A, Vittinghoff E, Ursell P, Connolly A, Olgin JE, Wong JK, Hsue PY (2021). Sudden cardiac death and myocardial fibrosis, determined by autopsy, in persons with HIV. N Engl J Med.

[39] Petersen SE, Friedrich MG, Leiner T, Elias MD, Ferreira VM, Fenski M, Flamm SD, Fogel M, Garg R, Halushka MK (2022). Cardiovascular magnetic resonance for patients With COVID-19. JACC Cardiovasc Imaging.

[40] Robbertse P, Doubell A, Innes S, Lombard C, Herbst P. Pulse wave velocity demonstrates increased aortic stiffness in newly diagnosed, antiretroviral naïve HIV infected adults: A case-control study. Medicine. 2022; Article in press. 10.1097/MD.0000000000029721 PMC9410660 36042673

[41] Lilian RR, Rees K, Mabitsi M, McIntyre JA, Struthers HE, Peters RPH (2019). Baseline CD4 and mortality trends in the South African human immunodeficiency virus programme: analysis of routine data. S Afr J HIV Med.

