# Supplementary material for: Altered cardiac structure and function in newly diagnosed people living with HIV: a prospective cardiovascular magnetic resonance study after the initiation of antiretroviral treatment
Source: Int J Cardiovasc Imaging. 2022 Aug 26;39(1):169–82. doi: 10.1007/s10554-022-02711-y (PMC9412796; doi:10.1007/s10554-022-02711-y)
Supplement: Supplementary file 1 — Supplementary file1 (DOCX 42 KB) [file 10554_2022_2711_MOESM1_ESM.docx]

**Appendix A: Bivariate analysis**

Considering correlations within the HIV infected group at both the baseline and follow-up visit (132 data points):

**LVEF**

The LVEF demonstrated a moderate positive correlation with the RVEF (r_s_=0.5; p<0.001). A low negative correlation was seen with LVEDV (r_s_=-0.3; p=0.003), averaged SI (r_s_=-0.2; p=0.004), creatinine (r_s_=-0.2; p=0.03) and triglycerides (r_s_=-0.2; p=0.05). No significant correlation was demonstrable between LVEF and age, TB disease, BMI, hsCRP, HIV viral load, CD4 count, eGFR, ethanol consumption, TR velocity, or SARS CoV-2 antibodies.

**RVEF**

The RVEF demonstrated moderate negative correlation with maximal LV thickness (r_s_=-0.4; p<0.001). A low positive correlation was present between the averaged E prime (r_s_=0.3; p=0.01). Low negative correlation was shown between RVEF and RVEDV (r_s_=-0.3; p=0.002), LV mass (r_s_=-0.3; p=0.002), averaged SI (r_s_=-0.2; p=0.04), creatinine (r_s_=-0.2; p=0.04), and ethanol consumption (r_s_=-0.2; p=0.01). No significant correlation was demonstrable between RVEF and age, TB disease, BMI, hsCRP, HIV viral load, CD4 count, eGFR, TR velocity, or SARS CoV-2 antibodies.

**LVEDV**

The LVEDV showed high positive correlation with the RVEDV and LV mass (r_s_=0.9 and 0.8; p<0.001). The LVEDV demonstrated a moderately positive correlation with the maximum LV thickness (r_s_=0.5), 6-minute walk distance (r_s_=0.5), haematocrit (r_s_=0.5), and creatine (r_s_=0.4; p values<0.001). A moderate negative correlation was seen with the resting heart rate (r_s_=-0.5; p<0.001). Additionally, the LVEDV showed low negative correlation with the WHO HIV clinical staging (r_s_=-0.3; p=0.001). A low positive correlation was seen with the CD4 count (r_s_=0.2; p=0.03), ethanol consumption (r_s_=0.3; p<0.001), TR velocity (r_s_=0.3; p=0.02), and mean arterial pressure (r_s_=0.2; p=0.02). Specifically, no correlation was demonstrable with eGFR.

**RVEDV**

The RVEDV demonstrated moderate positive correlation with the 6-minute walk distance (r_s_=0.5; p<0.001), haematocrit (r_s_=0.5; p<0.001), ethanol consumption (r_s_=0.4; p<0.001) and TR velocity (r_s_=0.3; p=0.003). The CD4 count (r_s_=0.3; p<0.001) and mean arterial pressure(r_s_=0.2; p<0.01) showed low positive correlation with RVEDV. Low negative correlation with the WHO HIV clinical staging (r_s_=-0.2; p=0.006) was present. Specifically, no correlation was demonstrable with eGFR.

**LV mass**

LV mass showed high positive correlation with the RVEDV (r_s_=0.8; p<0.001). A moderate positive correlation was present with basal longitudinal strain (r_s_=0.4; p=0.002), 6-minute walk test distance (r_s_=0.5; p<0.001), ethanol use (r_s_=0.5; p<0.001). A moderate negative correlation was seen with the WHO clinical stage (r_s_=-0.3; p<0.001). A low positive correlation was seen with CD4 count (r_s_=0.2; p=0.03), mean arterial pressure (r_s_=0.3; p<0.001), and absolute eGFR (r_s_=0.2; p=0.03).

**HIV viral load**

The HIV viral load (log) showed moderate negative correlation with the CD4 count (r_s_=-0.4; p<0.001), HDL cholesterol (r_s_=-0.3; p<0.001), triglycerides (r_s_=-0.3; p<0.001) and fasting blood glucose (r_s_=-0.2; p=0.006). HIV viral load demonstrated a low positive correlation with hsCRP (r_s_=0.3; p=0.002), and a low negative correlation with LA area (r_s_=-0.2; p=0.006).

Appendix A abbreviations:

LVEF = left ventricular ejection fraction; HIV = Human immunodeficiency virus; RVEF = right ventricular ejection fraction; LVEDV = left ventricular end diastolic volume; SI = left ventricular sphericity index; TB = tuberculous disease; BMI = body mass index; hsCRP =high sensitivity C-reactive protein; eGFR = estimated glomerular filtration rate; TR = tricuspid regurgitation; SARS CoV-2 = Severe acute respiratory syndrome coronavirus-2; RVEDV = right ventricular end diastolic volume; WHO = World Health Organisation; LA = left atrium

**Appendix B: Left ventricular ejection fraction linear regression analysis**

| **Model Summary** | | | | |
| --- | --- | --- | --- | --- |
|  | R | R Square | Adjusted R Square | Std. Error of the Estimate |
|  | .397^a^ | .158 | .096 | 5.476 |
| a. Predictors: (Constant), Current Tuberculous disease (yes), Ethanol units per week, Black African (yes), Age in years, HIV Infected (yes), Female (yes) | | | | |

| **ANOVA^a^** | | | | | | |
| --- | --- | --- | --- | --- | --- | --- |
|  | | Sum of Squares | df | Mean Square | F | Sig. |
|  | Regression | 455.803 | 6 | 75.967 | 2.533 | .027^b^ |
|  | Residual | 2429.095 | 81 | 29.989 |  |  |
|  | Total | 2884.898 | 87 |  |  |  |
| a. Dependent Variable: Left ventricular ejection fraction (%) | | | | | | |
| a. Predictors: (Constant), Current Tuberculous disease (yes), Ethanol units per week, Black African (yes), Age in years, HIV Infected (yes), Female (yes) | | | | | | |

| **Coefficients^a^** | | | | | | | | |
| --- | --- | --- | --- | --- | --- | --- | --- | --- |
|  | | Unstandardized Coefficients | | Standardized Coefficients | t | Sig. | 95.0% Confidence Interval for B | |
|  |  | B | Std. Error | Beta |  |  | Lower Bound | Upper Bound |
|  | (Constant) | 57.390 | 4.821 |  | 11.903 | .000 | 47.797 | 66.983 |
|  | Age in years | -.046 | .079 | -.061 | -.578 | .565 | -.204 | .112 |
|  | Female (yes) | 3.199 | 1.314 | .279 | 2.434 | .017 | .584 | 5.815 |
|  | Black African (yes) | -.971 | 1.382 | -.077 | -.703 | .484 | -3.721 | 1.778 |
|  | HIV Infected (yes) | -3.331 | 1.504 | -.252 | -2.215 | .030 | -6.323 | -.339 |
|  | Ethanol units per week | .050 | .048 | .120 | 1.029 | .307 | -.046 | .145 |
|  | Current Tuberculous disease (yes) | 3.037 | 1.914 | .168 | 1.586 | .117 | -.772 | 6.845 |
| a. Dependent Variable: Left ventricular ejection fraction (%) | | | | | | | | |

**Appendix C: Left ventricular ejection fraction linear regression analysis**

| **Model Summary** | | | | |
| --- | --- | --- | --- | --- |
|  | R | R Square | Adjusted R Square | Std. Error of the Estimate |
|  | .397^a^ | .158 | .096 | 5.476 |
| a. Predictors: (Constant), Current Tuberculous disease (yes), Ethanol units per week, Black African (yes), Age in years, HIV Infected (yes), Female (yes) | | | | |

| **ANOVA^a^** | | | | | | |
| --- | --- | --- | --- | --- | --- | --- |
|  | | Sum of Squares | df | Mean Square | F | Sig. |
|  | Regression | 455.803 | 6 | 75.967 | 2.533 | .027^b^ |
|  | Residual | 2429.095 | 81 | 29.989 |  |  |
|  | Total | 2884.898 | 87 |  |  |  |
| a. Dependent Variable: Left ventricular ejection fraction (%) | | | | | | |
| a. Predictors: (Constant), Current Tuberculous disease (yes), Ethanol units per week, Black African (yes), Age in years, HIV Infected (yes), Female (yes) | | | | | | |

| **Coefficients^a^** | | | | | | | | |
| --- | --- | --- | --- | --- | --- | --- | --- | --- |
|  | | Unstandardized Coefficients | | Standardized Coefficients | t | Sig. | 95.0% Confidence Interval for B | |
|  |  | B | Std. Error | Beta |  |  | Lower Bound | Upper Bound |
|  | (Constant) | 57.390 | 4.821 |  | 11.903 | .000 | 47.797 | 66.983 |
|  | Age in years | -.046 | .079 | -.061 | -.578 | .565 | -.204 | .112 |
|  | Female (yes) | 3.199 | 1.314 | .279 | 2.434 | .017 | .584 | 5.815 |
|  | Black African (yes) | -.971 | 1.382 | -.077 | -.703 | .484 | -3.721 | 1.778 |
|  | HIV Infected (yes) | -3.331 | 1.504 | -.252 | -2.215 | .030 | -6.323 | -.339 |
|  | Ethanol units per week | .050 | .048 | .120 | 1.029 | .307 | -.046 | .145 |
|  | Current Tuberculous disease (yes) | 3.037 | 1.914 | .168 | 1.586 | .117 | -.772 | 6.845 |
| a. Dependent Variable: Left ventricular ejection fraction (%) | | | | | | | | |

**Appendix D: Left ventricular end diastolic volume indexed to height linear regression analysis**

| **Model Summary** | | | | |
| --- | --- | --- | --- | --- |
|  | R | R Square | Adjusted R Square | Std. Error of the Estimate |
|  | .626^a^ | .392 | .346 | 12.453 |
| a. Predictors: (Constant), Current Tuberculous disease (yes), Ethanol units per week, Black African (yes), Age in years, HIV Infected (yes), Female (yes) | | | | |

| **ANOVA^a^** | | | | | | |
| --- | --- | --- | --- | --- | --- | --- |
|  | | Sum of Squares | df | Mean Square | F | Sig. |
|  | Regression | 8083.320 | 6 | 1347.220 | 8.687 | .000^b^ |
|  | Residual | 12562.123 | 81 | 155.088 |  |  |
|  | Total | 20645.443 | 87 |  |  |  |
| a. Dependent Variable: Left ventricular end diastolic volume indexed to height (ml/m) | | | | | | |
| a. Predictors: (Constant), Current Tuberculous disease (yes), Ethanol units per week, Black African (yes), Age in years, HIV Infected (yes), Female (yes) | | | | | | |

| **Coefficients^a^** | | | | | | | | | |
| --- | --- | --- | --- | --- | --- | --- | --- | --- | --- |
|  | | Unstandardized Coefficients | | Standardized Coefficients | t | Sig. | 95.0% Confidence Interval for B | |  |
|  |  | B | Std. Error | Beta |  |  | Lower Bound | Upper Bound |  |
|  | (Constant) | 127.610 | 10.965 |  | 11.638 | .000 | 105.794 | 149.426 |  |
|  | Age in years | -.103 | .180 | -.051 | -.572 | .569 | -.462 | .256 |  |
|  | Female (yes) | -17.191 | 2.989 | -.560 | -5.751 | .000 | -23.139 | -11.243 |  |
|  | Black African (yes) | -2.520 | 3.142 | -.075 | -.802 | .425 | -8.773 | 3.732 |  |
|  | HIV Infected (yes) | 7.544 | 3.420 | .213 | 2.206 | .030 | .739 | 14.348 |  |
|  | Ethanol units per week | -.027 | .109 | -.025 | -.247 | .805 | -.245 | .191 |  |
|  | Current Tuberculous disease (yes) | -13.863 | 4.353 | -.287 | -3.185 | .002 | -22.524 | -5.202 |  |
| a. Dependent Variable: Left ventricular end diastolic volume indexed to height (ml/m) | | | | | | | | | |

**Appendix E: Right ventricular end diastolic volume indexed to height linear regression analysis**

| **Model Summary** | | | | |
| --- | --- | --- | --- | --- |
|  | R | R Square | Adjusted R Square | Std. Error of the Estimate |
|  | .535^a^ | .286 | .233 | 15.445 |
| a. Predictors: (Constant), Current Tuberculous disease (yes), Ethanol units per week, Black African (yes), Age in years, HIV Infected (yes), Female (yes) | | | | |

| **ANOVA^a^** | | | | | | |
| --- | --- | --- | --- | --- | --- | --- |
|  | | Sum of Squares | df | Mean Square | F | Sig. |
|  | Regression | 7751.671 | 6 | 1291.945 | 5.416 | .000^b^ |
|  | Residual | 19321.601 | 81 | 238.538 |  |  |
|  | Total | 27073.273 | 87 |  |  |  |
| a. Dependent Variable: Right ventricular end diastolic volume indexed to height EDV (ml/m) | | | | | | |
| a. Predictors: (Constant), Current Tuberculous disease (yes), Ethanol units per week, Black African (yes), Age in years, HIV Infected (yes), Female (yes) | | | | | | |

| **Coefficients^a^** | | | | | | | | |
| --- | --- | --- | --- | --- | --- | --- | --- | --- |
|  | | Unstandardized Coefficients | | Standardized Coefficients | t | Sig. | 95.0% Confidence Interval for B | |
|  |  | B | Std. Error | Beta |  |  | Lower Bound | Upper Bound |
|  | (Constant) | 104.514 | 13.598 |  | 7.686 | .000 | 77.458 | 131.570 |
|  | Age in years | -.016 | .224 | -.007 | -.070 | .944 | -.461 | .430 |
|  | Female (yes) | -15.681 | 3.707 | -.446 | -4.230 | .000 | -23.057 | -8.305 |
|  | Black African (yes) | -.494 | 3.897 | -.013 | -.127 | .899 | -8.248 | 7.260 |
|  | HIV Infected (yes) | 9.920 | 4.241 | .245 | 2.339 | .022 | 1.481 | 18.359 |
|  | Ethanol units per week | .058 | .136 | .046 | .429 | .669 | -.212 | .328 |
|  | Current Tuberculous disease (yes) | -6.756 | 5.399 | -.122 | -1.251 | .214 | -17.497 | 3.986 |
| a. Dependent Variable: Right ventricular end diastolic volume indexed to height EDV (ml/m) | | | | | | | | |

**Appendix F: Left ventricular mass indexed to height linear regression analysis**

| **Model Summary** | | | | |
| --- | --- | --- | --- | --- |
|  | R | R Square | Adjusted R Square | Std. Error of the Estimate |
|  | .666^a^ | .444 | .403 | 9.843 |
| a. Predictors: (Constant), Current Tuberculous disease (yes), Female (yes), Black African (yes), Age in years, Absolute eGFR, HIV Infected (yes) | | | | |

| **ANOVA^a^** | | | | | | |
| --- | --- | --- | --- | --- | --- | --- |
|  | | Sum of Squares | df | Mean Square | F | Sig. |
|  | Regression | 6272.084 | 6 | 1045.347 | 10.789 | .000^b^ |
|  | Residual | 7847.746 | 81 | 96.886 |  |  |
|  | Total | 14119.830 | 87 |  |  |  |
| 1. Left ventricular mass indexed to height (g/m) | | | | | | |
| 1. Predictors: (Constant), Current Tuberculous disease (yes), Female (yes), Black African (yes), Age in years, Absolute estimated glomerular filtration rate (ml/min/1.73m^2^), HIV Infected (yes) | | | | | | |

| **Coefficients^a^** | | | | | | | | |
| --- | --- | --- | --- | --- | --- | --- | --- | --- |
|  | | Unstandardized Coefficients | | Standardized Coefficients | t | Sig. | 95.0% Confidence Interval for B | |
|  |  | B | Std. Error | Beta |  |  | Lower Bound | Upper Bound |
|  | (Constant) | 64.471 | 8.913 |  | 7.233 | .000 | 46.736 | 82.205 |
|  | Age in years | .162 | .141 | .097 | 1.148 | .254 | -.119 | .443 |
|  | Female (yes) | -14.802 | 2.135 | -.583 | -6.932 | .000 | -19.051 | -10.554 |
|  | Black African (yes) | -.021 | 2.517 | -.001 | -.008 | .993 | -5.029 | 4.986 |
|  | HIV Infected (yes) | 5.987 | 2.823 | .205 | 2.121 | .037 | .370 | 11.604 |
|  | Absolute eGFR (ml/min/1.73m^2^) | .178 | .040 | .394 | 4.410 | .000 | .098 | .258 |
|  | Current Tuberculous disease (yes) | -6.279 | 3.437 | -.157 | -1.827 | .071 | -13.117 | .560 |
| a. Dependent Variable: Left ventricular mass indexed to height (g/m) | | | | | | | | |

eGFR = Estimated glomerular filtration rate
